# Microfluidics and Metabolomics Reveal Symbiotic Bacterial–Fungal Interactions Between *Mortierella elongata* and *Burkholderia* Include Metabolite Exchange

**DOI:** 10.3389/fmicb.2019.02163

**Published:** 2019-10-01

**Authors:** Jessie K. Uehling, Matthew R. Entler, Hannah R. Meredith, Larry J. Millet, Collin M. Timm, Jayde A. Aufrecht, Gregory M. Bonito, Nancy L. Engle, Jessy L. Labbé, Mitchel J. Doktycz, Scott T. Retterer, Joseph W. Spatafora, Jason E. Stajich, Timothy J. Tschaplinski, Rytas J. Vilgalys

**Affiliations:** ^1^Department of Botany and Plant Pathology, Oregon State University, Corvallis, OR, United States; ^2^Department of Biology, Duke University, Durham, NC, United States; ^3^Biosciences Division, Oak Ridge National Laboratory, Oak Ridge, TN, United States; ^4^Department of Epidemiology, Johns Hopkins Bloomberg School of Public Health, Baltimore, MD, United States; ^5^Department of Biomedical Engineering, Duke University, Durham, NC, United States; ^6^The Bredesen Center, The University of Tennessee, Knoxville, Knoxville, TN, United States; ^7^Department of Plant, Soil and Microbial Sciences, Michigan State University, East Lansing, MI, United States; ^8^Genome Science & Technology, The University of Tennessee, Knoxville, Knoxville, TN, United States; ^9^Center for Nanophase Materials Sciences, Oak Ridge National Laboratory, Oak Ridge, TN, United States; ^10^Department of Microbiology and Plant Pathology, Institute for Integrative Genome Biology, University of California, Riverside, Riverside, CA, United States

**Keywords:** bacterial–fungal interactions, microfluidics, metabolomics, fatty acid, symbiotic evolution, *Mortierella elongata*, *Burkholderia*, plant associated microbes

## Abstract

We identified two poplar (*Populus* sp.)-associated microbes, the fungus, *Mortierella elongata* strain AG77, and the bacterium, *Burkholderia* strain BT03, that mutually promote each other’s growth. Using culture assays in concert with a novel microfluidic device to generate time-lapse videos, we found growth specific media differing in pH and pre-conditioned by microbial growth led to increased fungal and bacterial growth rates. Coupling microfluidics and comparative metabolomics data results indicated that observed microbial growth stimulation involves metabolic exchange during two ordered events. The first is an emission of fungal metabolites, including organic acids used or modified by bacteria. A second signal of unknown nature is produced by bacteria which increases fungal growth rates. We find this symbiosis is initiated in part by metabolic exchange involving fungal organic acids.

## Introduction

Fungi and bacteria regularly co-occur, and engage in symbioses that affect many facets of human health and life (Hogan and Kolter, 2002; Frey-Klett et al., 2011; Labbé et al., 2014; Wolfe and Dutton, 2014; Deveau et al., 2018). Bacterial–fungal interactions (BFIs) frequently occur in the rhizosphere, or vicinity of actively growing plant roots, and sometimes stimulate each other’s growth and benefit plant health (Frey-Klett et al., 2007; Shakya et al., 2013; Deveau et al., 2018). Whether BFIs lead to mutualism, commensalism or antagonism is dependent on abiotic and biotic factors such as resource availability and population densities (Deveau et al., 2016). Fungi respond to bacteria in several ways including alterations in fungal metabolism, cell wall composition, and growth (Deveau et al., 2007; Schroeckh et al., 2009; Jonkers et al., 2010; Mela et al., 2011; Mathioni et al., 2013; Ipcho et al., 2016; Salvioli et al., 2016). Although many bacterial–fungal symbioses are documented, details of the interactions preceding these symbioses remain elusive, and the signals involved uncharacterized. To understand how symbiotic relationships are established and maintained, we need to understand the dynamics and conditionality of how bacteria and fungi produce, transmit, receive, and interpret signals from one another (Putnoky et al., 1988; Isack and Reyer, 1989; Gagnon and Ibrahim, 1998; Nadal and Paszkowski, 2013).

Trophic trades and pre-symbiotic signal exchanges are integral parts of symbiotic establishment, including in the inter-kingdom symbioses that profoundly modified our current world. Symbioses involving trophic interactions include chloroplasts and mitochondria (Gray et al., 1999), and the gut microbiomes of animals and insects (Moran et al., 2005; Hess et al., 2011). These symbioses depend on the remarkable capacity for two, often highly diverged, free-living organisms to accurately produce, transmit and interpret signals from one another (Putnoky et al., 1988; Isack and Reyer, 1989; Gagnon and Ibrahim, 1998; Nadal and Paszkowski, 2013). In some instances pre-symbiotic signal exchanges are conditional (Moné et al., 2014; Deveau et al., 2016), highlighting the importance of identifying not only the signals involved, but also the contexts that facilitate successful symbiosis initiation.

Our research group has an extensive culture collection of genotyped, plant beneficial bacteria and fungi (Shakya et al., 2013; Labbé et al., 2014; Timm et al., 2016). The bacteria and fungi described here were isolated from and regularly inhabit the poplar (*Populus*) rhizosphere. Together with their plant hosts they form a multi-trophic beneficial network (Labbé et al., 2014). These co-occurring symbiotic bacteria and fungi present a compelling system to study early events in the initiation of symbioses because they are genotyped and culturable in the laboratory, and they offer quantifiable symbiotic phenotypes such as growth rate shifts. Despite these benefits, studying BFIs is challenging in that precisely monitoring bacterial and fungal growth requires multiple approaches. For bacteria, changes in optical density over time in liquid cultures and plate-based colony counts are used to quantify growth rates (Contois, 1959). In contrast, for fungi quantification efforts include measuring radial colony expansion rates on agar plates; counting spores or colony forming units; quantitative PCR (qPCR); and labeling cell wall components such as ergosterols (Gessner et al., 1991; Hierro et al., 2006; Quecine et al., 2017). Fungal growth rate quantification on plates lacks the sensitivity to detect unique behaviors of single hyphae due to averaging colony expansion rates (Trinci, 1969), and thus potentially loses valuable data on fungal physiology. Microfluidics and time lapse microscopy enable the confinement of individual hyphae and interacting bacteria or molecules allowing for microscopic measurements and controlled manipulation of molecular symbiotic environments (Hanson et al., 2006; Moore et al., 2008; Held et al., 2010; Agudelo et al., 2013; Aspray et al., 2013; Berthier et al., 2013; Stanley et al., 2014; Massalha et al., 2017; Fuchs et al., 2019; Schmieder et al., 2019; Tayyrov et al., 2019).

Here, we characterize pre-symbiotic dynamics between the poplar associates *Mortierella elongata* (Mortierellales, Fungi) and *Burkholderia* (Burkholderiales, Betaproteobacteria) using plate and liquid based growth assays coupled with time lapse videography and comparative metabolomics. Our research goals were two-fold: first to identify conditions that induce pre-symbiotic fungal growth stimulation; and second to elucidate the quantities, specificity, and directionality of molecules exchanged before symbiosis is initiated. To better understand pre-symbiotic interaction dynamics, we quantified microbial growth rates on plates, in conditioned liquid media using time-lapse microfluidic videos, and by monitoring optical densities in liquid cultures. Through our microfluidic growth quantification data, we were able to identify conditions that promote fungal growth. We then quantified metabolite accumulation in implicated media compared to one another. Conditions conducive to establishing BFIs and metabolites implicated in the *M. elongata* – *Burkholderia* symbiosis are discussed.

## Results

### Co-culturing *Burkholderia* With *M. elongata* Increases Fungal and Bacterial Growth Rates

**FIGURE 1:**
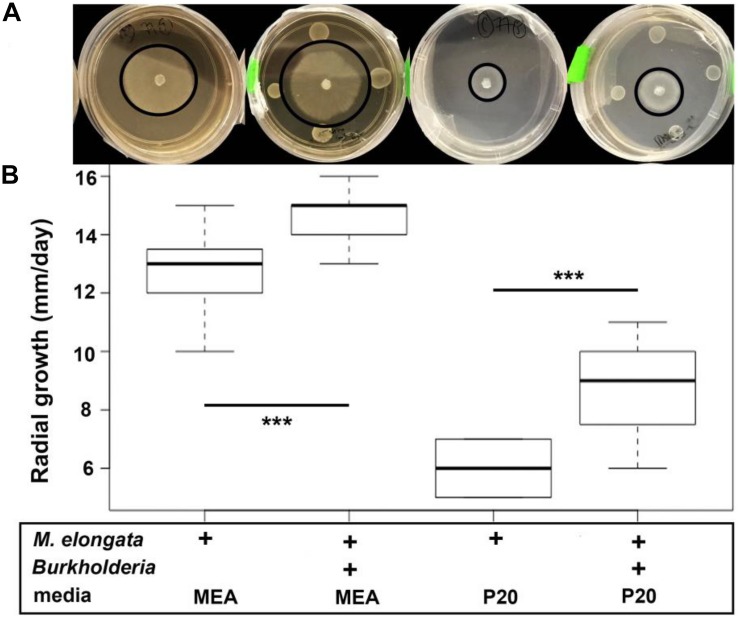
*M. elongata* and *Burkholderia* co-culture experiments and radial growth rate data from plate-based co-culture experiments. Experiments performed on malt extract agar (MEA, dark) and P20 (light) media. In each image, the perimeter of the fungal colony is outlined in black. **(A)** *M. elongata* grown in monoculture and co-culture with *Burkholderia* on solid P20 and MEA media. **(B)** Radial growth rates in mm/day. Box plots correspond to plate images above from left to right for *M. elongata* without and with *Burkholderia* on P20 and MEA media. ^∗∗∗^*p* < 0.005, Student’s *t*-tests.

**FIGURE 2:**
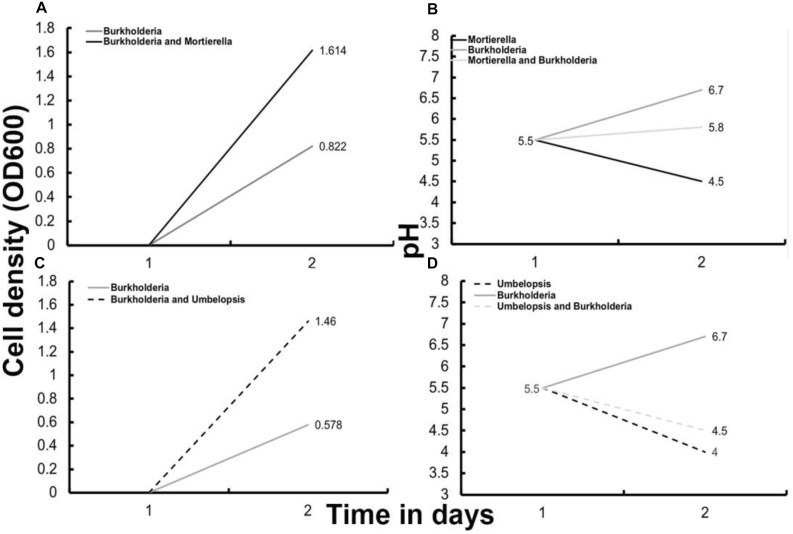
Optical density based growth rates **(A,C)** and pH **(B,D)** after 48 h of bacterial growth with and without fungi in liquid P20 media. **(A)** Optical density growth data for *Burkholderia* cultured with and without *M. elongata*. **(B)** Media pH for *Burkholderia* cultured with and without *M. elongata* and *M. elongata* alone. **(C)** Optical density growth data for *Burkholderia* cultured with and without *Umbelopsis*. **(D)** Media pH for *Burkholderia* cultured with and without *Umbelopsis* and for *Umbelopsis* cultured alone.

Microbial growth rate assays were performed with two beneficial *Populus* root associates, *Burkholderia* strain BT03 and *M. elongata* strain AG77 (Table 1) (hence forth called *Burkholderia* and *M. elongata*, respectively). The possibility that the observed growth rate increases were related to a starvation response was tested by comparing radial growth rates on rich (malt extract agar, MEA) and poor (Pachlewski, P20) media as derived from Figure 1. The radial growth rate of *M. elongata* on P20 plates increased from 3.00 ± 0.43 mm/day in mono-culture to 4.38 ± 0.74 mm/day when co-cultured with *Burkholderia* (*p* = 7.099e-04 Student’s *t-*tests) (Figure 1A). A similar trend was observed on MEA plates, with radial growth rate increasing from 6.41 ± 1.27 mm/day (mono-culture) to 7.29 ± 0.39 mm/day (co-culture, *p* = 3.059e-05) (Figure 1B and Table 2). Likewise, *Burkholderia’s* unbuffered P20 liquid culture population carrying capacity increased from 0.8822 OD600 in mono-culture to 1.614 OD600 co-culture with *M. elongata* (Figure 2A). Likewise, *Burkholderia’s* maximum population density in unbuffered P20 liquid culture increased from 0.8822 OD600 in mono-culture to 1.614 OD600 co-culture with *M. elongata* (Figure 2A).

**TABLE 1:** Microbial strains and accession numbers for isolates analyzed in microfluidic, optical density, and plate-based assays.

**Kingdom**	**Phylum**	**Genus**	**Strain**	**NCBI accession**
Fungi	Mortierellomycotina	*Mortierella elongata*	AG77	KP772747
Bacteria	Betaproteobacteria	*Burkholderia* sp.	BT03	Go0012187
Fungi	Mortierellomycotina	*Umbelopsis*	PMI	n/a
Bacteria	Betaproteobacteria	*Methylibium*	PMI	NZ_CP029606.1

**TABLE 2:** Fungal growth rates of *M. elongata* grown in fresh and conditioned media from radial growth and microfluidic assays.

**Strain**	**Media**	**pH**	**Conditions**	**Rate**		**Data source**	
					
				**uM/min**	**mM/day**	**Radial growth**	**Microfluidics**
*M. elongata* AG77+	P20	N/A	77 +	3.3 ± 0.58	(4.75 ± 0.84)		×
*M. elongata* AG77−	P20	N/A	77-	3.89 ± 0.69	(5.60 ± 0.99)		×
*M. elongata*	P20	N/A	BSP	3.8 ± 1.16	(5.47 ± 1.7)		×
*M. elongata*	P20	N/A	DC	4.67 ± 0.91	(6.72 ± 1.31)		×
*M. elongata*	P20	4.5	4.5 BSP	3.57 ± 0.70	(5.14 ± 1.0)		×
*M. elongata*	P20	N/A	N/A	(2.16 ± 0.40)	3.00 ± 0.43	×	
*M. elongata*	P20	N/A	Co-culture with *Burkholderia*	(3.03 ± 0.49)	4.38 ± 0.74	×	
*M. elongata*	MEA	N/A	N/A	(4.46 ± 0.44)	6.41 ± 0.63	×	
*M. elongata*	MEA	N/A	Co-culture with *Burkholderia*	(5.06 ± 0.28)	7.29 ± 0.39	×	

*Parentheses indicate rates that are extrapolated for comparison. ± Indicate standard deviations.*

### Signaling Between *M. elongata* and *Burkholderia* Is Bi-Directional and Multi-Phased

**FIGURE 3:**
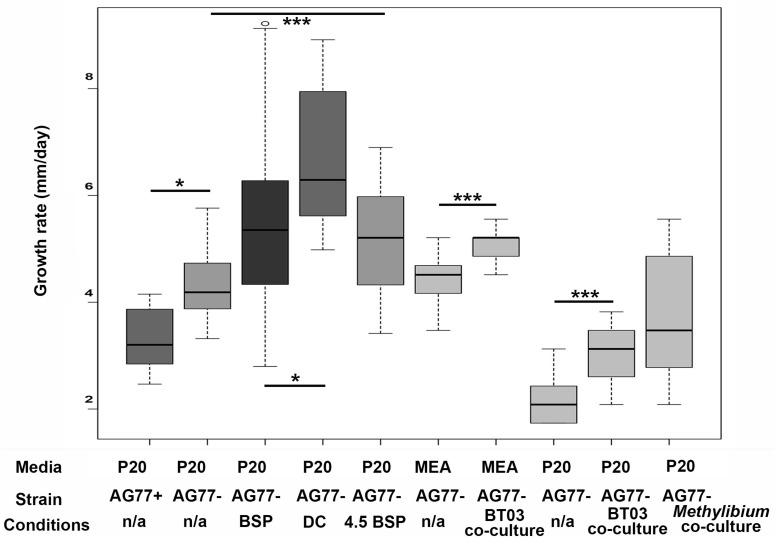
Fungal growth rates of *M. elongata* in response to conditioned media from microfluidic and plate based growth assays. All values extrapolated to units of millimeters growth per day. Growth data from microfluidic device data are indicated in gray scale boxes on the left (see Table 3). AG77+/– refer to endosymbiont presence and absence used in device validation. BSP media was conditioned with bacterial growth, DC media was conditioned with successive fungal then bacterial growth, and pH 4.5 BSP media was buffered at pH.5 and conditioned by bacterial growth. Data from plates are in lighter gray boxes on the right and are coded by media type. ^∗^*p* ≤ 0.05, ^∗∗∗^*p* ≤ 0.001.

**FIGURE 4:**
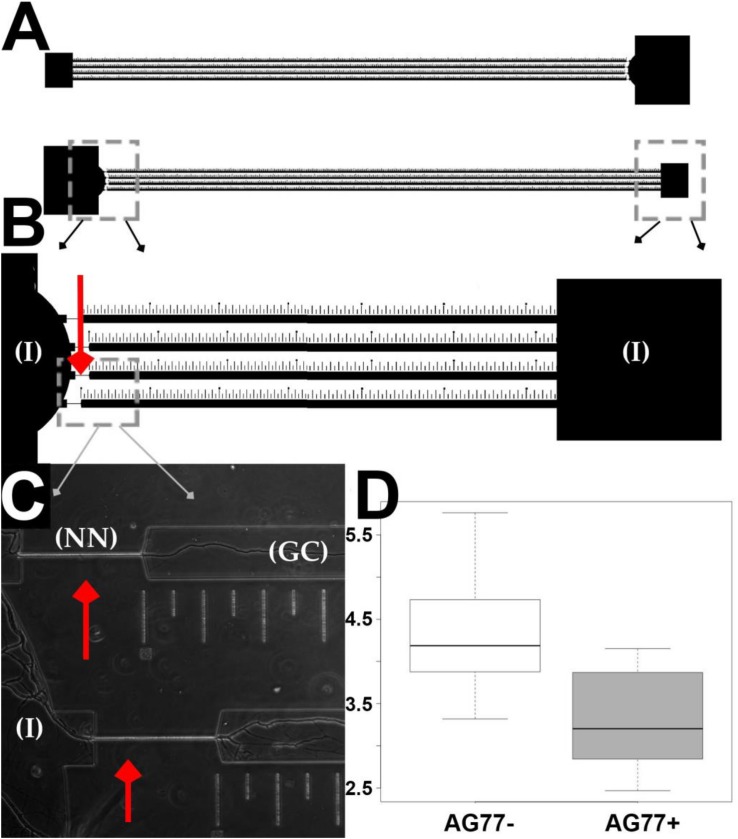
Microfluidic device design and validation of fungal growth rate data. **(A)** Two chambers running parallel on a mounted slide. **(B)** Narrow necks indicated by red arrows running from inoculation chambers (I) to growth chamber etched with ruler in micrometers for single hyphal growth quantification. **(C)** *M. elongata* growing through narrow necks (NN) from inoculation chambers (I) into growth chamber (GC), imaged from device mounted onto a slide and filled with P20 liquid media. **(D)** Comparative microfluidic growth rate data (extrapolated to mm/day) for *M. elongata* with and without *Mycoavidus cysteinexigens* (AG77+ and AG77–).

Plate-based co-culture and optical density-based assays demonstrated that *Burkholderia* and *M. elongata* stimulate each other’s growth but did not provide precise measurements of individual hyphal responses nor any indication for the directionality of the signaling events involved. Time-lapse videos of co-culture experiments captured hyphal growth rate dynamics preceding microbial colony contact on MEA plates. The videos show that just before microbial contact, fungal hyphae in colonies exhibited differential growth rates and some rapidly increased their growth rates (Supplementary Video S1). This indicates extracellular signal exchange and underscores the need to further study individual hyphae at the fungal colony perimeter for response to extracellular bacterial products. Given that this individual hyphal phenotypic variation was not detectable using the traditional radial growth assays on plates, we designed a microfluidic device that enabled highly precise hyphal growth measurements in liquid media (Figures 3, 4). We reasoned that if we observed a fungal growth increase in media conditioned by, but not containing live *Burkholderia* bacterial populations, single, directional signal exchanges involved could be identified and studied.

To identify the conditions that induce pre-symbiotic signal exchange and increased microbial growth rates, we quantified fungal growth rates in variously conditioned media using time-lapse videos from microfluidic devices (Table 3, Figure 4, and Supplementary Video S2). The observation that *M. elongata* growth rates increased in plate-based co-cultures with *Burkholderia* (Figure 1) led us to hypothesize that *Burkholderia* secretes growth-increasing signals into its surrounding environment that are perceived by *M. elongata*. To test this hypothesis, *Burkholderia* conditioned media from log phase cultures were generated, resulting in cell free supernatant referred to as bacterial conditioned media (bacterial spent or BSP), that was used to fill microfluidic devices and grow *M. elongata*. Fungal growth rates from the bacterial conditioned media (5.60 ± 0.99 mm/day) were compared to growth rates in the unconditioned P20 control (5.47 ± 1.7 mm/day) and a slight decrease was observed in BSP medium (*p* = 0.004913, Student’s *t*-test) (Figure 3 and Table 2). We then hypothesized that a signal from bacteria to fungi may be secondary to a preliminary signal from fungi to bacteria. When grown in the double conditioned (DC) media, *M. elongata*’s growth rate (6.72 ± 1.31 mm/day) increased significantly compared to the P20 control (5.47 ± 1.7 mm/day) (Figure 3 and Table 2). Together these data suggest that signaling is bi-directional and that the first signal exchange is from fungi to bacteria. Further these data show that a second diffusible bacterial signal is secreted that induces a fungal growth increase after bacteria sense fungal presence.

**TABLE 3:** Conditioning of media for fungal and bacterial growth rate experiments.

**Abbreviation**	**pH**	**Bacterial**	**Fungal**	**Co-culture**
	**buffered**	**growth**	**growth**	**growth**
P20	None	None	None	None
4.5	4.5	None	None	None
4.5 FSP	4.5	n/a	48 h	none
4.5 BFI	4.5	None	n/a	48 h
6.5	6.5	None	None	None
6.5 FSP	6.5	n/a	48 h	None
6.5 BFI	6.5	None	n/a	48 h
BSP	None	48 h	None	None
DC	None	48 h	48 h	None
4.5 BSP	4.5	48 h	None	None

*All media consisted of P20. Buffering and microbial growth conditioning are indicated. For media conditioned by microbial growth, isolates were grown for 48 h in media, then removed by centrifugation and 0.2 uM filtration.*

### Variation in *Burkholderia* Population Growth Associated With pH and Co-culture

**FIGURE 5:**
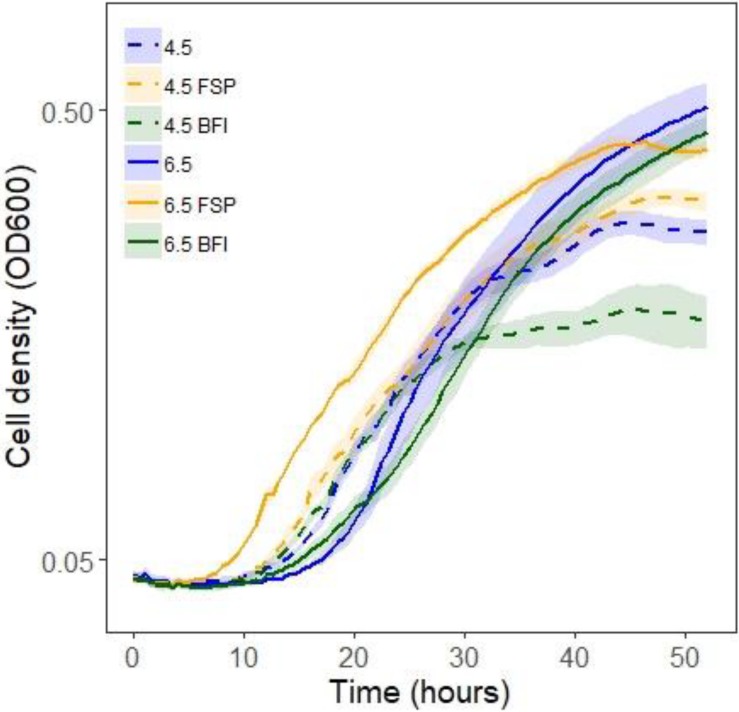
Optical density (OD600) growth data for *Burkholderia* growing in P20 media and P20 media conditioned by buffering and microbial growth (see legend and Table 3 for details). Line color and texture reflect pH and conditioning, solid and dashed lines reflect pH 6.5 and 4.5, respectively, while blue, yellow, and green lines indicate plain unconditioned media, fungal spent (FSP), and co-cultured bacterial fungal interaction (BFI) conditioned media, respectively.

Given that growth stimulation in this pair is bi-directional and media conditioned initially by fungi and then by bacteria (DC media) produced the greatest fungal growth rates, conditions affecting bacterial capacity for growth were investigated. The carrying capacity for *Burkholderia* cultures was measured via optical density in media across different pH and types of conditioning (Table 3). Bacterial population carrying capacities were quantified from OD data in liquid media buffered at constant pH and conditioned by microbial growth (Table 3). We evaluated buffered media at the fungal (4.5) and bacterial (6.5) pH optima in liquid P20 monoculture (Figure 2B). Comparing media, *Burkholderia* grew optimally in P20 media buffered at pH 6.5 with no conditioning (Figure 5). Media conditioned by co-culture and buffered at pH 4.5 (4.5 BFI) produced the lowest carrying capacity (Figure 5). Among the media buffered at pH 4.5, conditioning media with fungal monoculture growth (4.5 FSP) increased the carrying capacity of *Burkholderia* (Table 3 and Figure 5). Together these observations suggest pH 6.5 is optimal for *Burkholderia* growth and that extracellular fungal products increase the carrying capacity of *Burkholderia* when the pH is suboptimal.

### Pre-symbiotic Exchanges From Fungi to Bacteria Include Organic Acids

After the conditions that increase microbial growth rates were identified, the nature of the signals involved were investigated. The observation that fungal mono-cultures were more acidic than co-cultures (Figure 2) led us to hypothesize that pH or a molecule that modulates pH may be an initial signal from fungi to bacteria. To test this hypothesis, hyphal growth was measured in BSP media that was buffered to pH 4.5 (4.5 BSP). *M. elongata* grew more slowly in 4.5 BSP media (5.14 ± 1.0 mm/day), compared to unbuffered BSP media (5.47 ± 1.7 mm/day) (Figure 3 and Table 2). These data indicate that pH is not a direct signal, but that secreted molecules which alter pH during early symbiotic interactions are involved in symbiotic dynamics.

**FIGURE 6:**
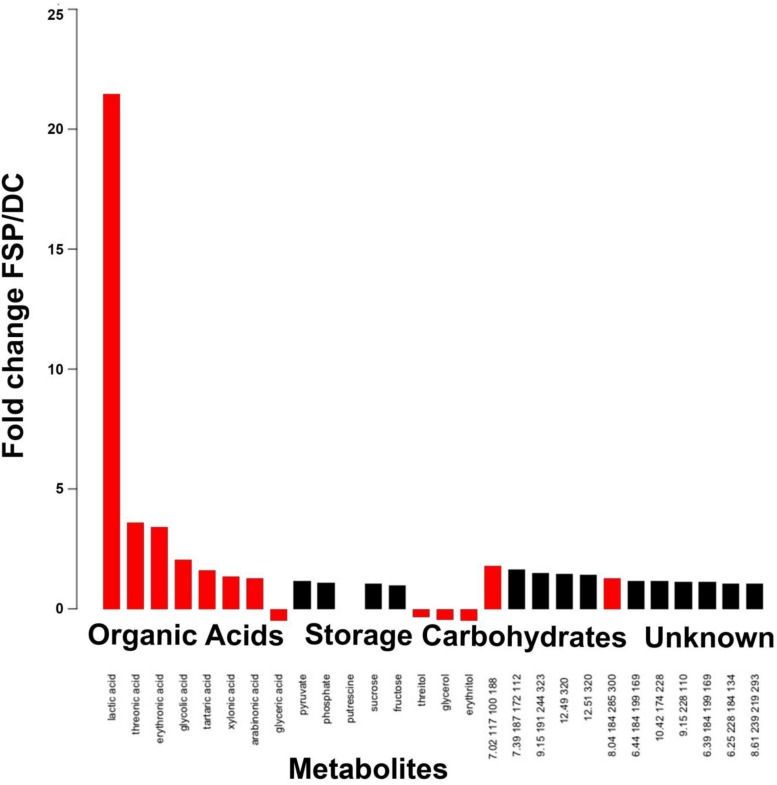
Metabolite accumulation in fungal conditioned (FSP) media compared to double conditioned (DC) media, see Table 3 for detail on conditioning. The accumulation of metabolites arranged by category are reported as fold change in FSP relative to DC media. Red bars indicate significance across replicates based on *p* < 0.05, Students *t*-test.

Given that *M. elongata* is a robust producer of lipids (Uehling et al., 2017), we hypothesized that lipids, fatty acids and other metabolites underpin this growth promoting interaction. To test this hypothesis, fungal conditioned (FSP) media (Table 3) was collected and metabolites were quantified. FSP media had only been conditioned by fungal growth, compared to DC media, which was conditioned first by fungal, then by bacterial growth (Supplementary Table S1). The FSP media were compared to DC and BFI media for metabolite accumulation. We found that fungal organic acids accumulate in excess (Figure 6, Supplementary Figures S1, S2, and Supplementary Table S1), indicating these products are used or modified by *Burkholderia* and potentially related to symbiotic growth phenotypes (Figure 6). For example, erythronic acid accumulated 8.44-fold and 3.42-fold in FSP media compared to the BFI media (Supplementary Figure S2) and DC media, respectively (Figure 6) (*p* < 0.05). We also observed lactic acid accumulated 21.47-fold and 2.88-fold in FSP media, relative to BFI and DC media, respectively (Figure 6, Supplementary Figures S1, S2, and Supplementary Table S1). There were also several unidentified metabolites that accumulated in the same conditions, which may be involved in the interactions (Figure 6 and Supplementary Figures S1, S2). Together these data suggest that multiple bi-directional signals are exchanged, and fungal metabolites are among the first potentially used as a trophic resource by bacteria.

### Bacterial–Fungal Interactions Between Betaproteobacteria and Mucoromycotina Fungi Share Common Features

To determine if our observations were generalizable to other BFIs, we analyzed the ability of close relatives isolated from the poplar rhizosphere to stimulate microbial growth and modulate media pH (Table 1). *Methylibium* (Comamonadaceae, Betaproteobacteria) is a bacterial *Populus* rhizosphere strain and a close relative of *Burkholderia* based on phylogenetic analysis of 16S rDNA and genome sequence data (Timm et al., 2016). *M. elongata* growth rates increased when grown in co-culture with *Methylibium* on solid plates (data not shown). Similarly, *Umbelopsis* (Umbelopsidaceae, Mucoromycota) is an oleaginous close relative of *Mortierella*, and is regularly isolated from plant rhizospheres (Shakya et al., 2013; Bonito et al., 2014). *Umbelopsis* increased the *Burkholderia* carrying capacity from 0.578 to 1.46 OD600 after 48 h in P20 liquid co-culture (Figure 2C). pH of liquid P20 media conditioned by *Umbelopsis* dropped from pH 4.5 to 4 (Figure 2D), suggesting that pH modification, potentially due to fatty acid secretion, is not unique to *M. elongata–Burkholderia* symbioses. Further, the observation that close relatives in the Mucoromycota and Betaproteobacteria have similar effects on one another and their surroundings may point to shared symbiotic mechanisms in these clades (Compant et al., 2008).

## Discussion

### Bacterial–Fungal Interactions Include Bi-Directional, Multiphase Signal Exchange

Our first research goal was to identify conditions which lead to *M. elongata* growth rate increases during co-culture with *Burkholderia*. By comparing fungal growth in various media, we identified conditions that increase fungal growth rates and characterized the potential signals exchanged. We found that DC media, which was conditioned by successive rounds of fungal, then bacterial growth, elicited highest fungal growth rates in the microfluidic device.

**FIGURE 7:**
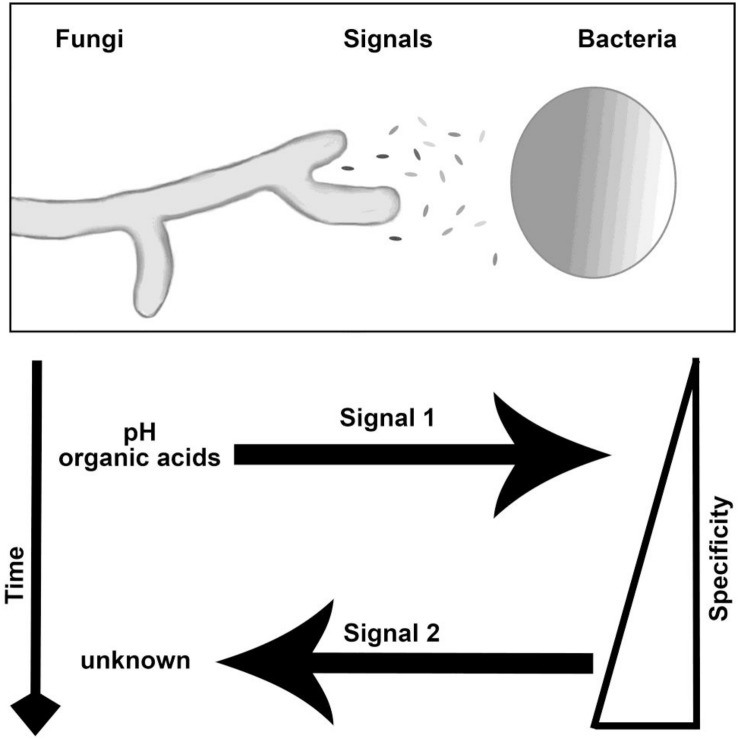
Interaction and signaling model for *M. elongata* and *Burkholderia*. The nature, timing, specificity, and direction of signals are indicated in the **lower panel**. Findings included within show that there are at least two ordered signals exchanged, first involving the secretion and utilization of fungal organic acids by bacteria and second the secretion of an undescribed factor by *Burkholderia* which stimulated fungal growth. Specificity refers to the ability of close relatives to induce similar morphological shifts, see Tables 2, 3 for more details.

Our study suggests *Burkholderia* emits a fungal growth inducing signal after detecting the presence of organic acids produced by *M. elongata* (Figure 7). The precision in microfluidic based fungal phenotype quantification allowed us to disentangle the order and direction of multiple signal exchange during early symbioses. These conclusions would have not been possible by analyzing time lapse videos of fungi and bacteria growing on plates. The novel combination of growth rate data from microfluidic devices, agar plates, and optical density from plate reader allows for assessment of unique aspects of signaling. To the best of our knowledge, this is the first application of microfluidics to study the dynamics of environmental microbial growth stimulation due to BFIs.

The nature of symbiotic interactions and the conditionality of fungal growth increases by bacterial contact have been investigated by Deveau et al. (2007, 2015, 2018). They found that in some instances *Pseudomonas fluorescens* increases growth and branching rates of *Laccaria bicolor*, while in others, specifically under iron limitation, the microbial partners become antagonistic toward each other (Deveau et al., 2016). This example of a bacterial–fungal pair is among many described as mycorrhizal helper bacteria systems (Frey-Klett et al., 2007).

Similar microfluidic approaches for studying antagonistic BFIs have been taken in examining fungal morphology and response to maze-like environments (Held et al., 2010; Stanley et al., 2014). Our device is novel in including sub-structures for the isolation of small amounts of tissue for imaging. Previous work examining BFI signaling dynamics has focused on fungal responses to bacterial signals (Deveau et al., 2007, 2016; Ipcho et al., 2016). Here, we dissected BFIs into multiple, phased signal exchanges and identified initial signals transmitted from fungi to bacteria.

We observed highly variable hyphal morphologies and growth rates on plate based assays. In the microfluidic channels, *M. elongata* no longer exhibited the same altered branching morphologies. Similar effects of hyphal confinement have been observed and discussed by Hanson et al. (2006). While strengths of the microfluidic approach include precision in hyphal growth rate measurements comparable to plate-based growth data, a drawback is a loss in the ability to measure branching phenotypes due to restricted directional growth compared to plate-based assays. However, hyphae encounter similar spatial constraints in the soil environment, potentially rendering plate-based growth assays superfluous. Similarly, Held et al. (2010) have studied and described fungal thigmotropism, the response of hyphae to the encountering obstacles, finding that confinement impacts fungal branching behaviors in *Neurospora crassa.* We found that variance in fungal growth rates was higher in the microfluidic devices compared to plate based assays. However, in each case the magnitude of the growth increase stayed nearly identical in the microfluidic devices and plates, indicating that we more precisely captured variance in individual hyphal behaviors.

Together these data suggest that microfluidics allow for accurate imaging and assessment of BFIs from growth rates from time-lapse videos, but that other approaches offer insight into phenotypes lost in microfluidics such as branching dynamics. Future approaches may circumvent these obstacles by incorporating a larger diameter chamber and by imaging hyphae in stacks. Microfluidic devices will also enable studies of fungal biology not addressed here including cytoplasmic streaming and intrahyphal endosymbiont movement. For instance, all Mucoromycota fungi lack regular cell walls, and many associate with Betaproteobacterial exo- and endosymbiotic bacteria which could be imaged using similar microfluidic device designs.

### Burkholderia Uses Fungal Organic Acids During Their Pre-symbiotic Interactions

Our second research goal was to characterize molecular signals transmitted between *M. elongata* and *Burkholderia* during symbiotic establishment. We observed that growth stimulation is bi-directional, and that media conditioned by growing and removing first *M. elongata*, then *Burkholderia* (DC media) most strongly stimulated fungal growth rates.

After implicating the highest fungal growth increase with the specific conditions used to produce DC media, we quantified metabolites in FSP media (conditioned only by fungal growth) and DC media (conditioned by successive fungal then bacterial growth). We observed that fatty acids and storage carbohydrates accumulated in FSP media. For example, erythronic and threonic acids accumulate by 3.42- and 3.58-fold in FSP compared to DC media. We also noted that *Burkholderia* exhibited a higher population carrying capacity in media that had been conditioned by fungal metabolites (FSP media).

We concluded that initial phases of this interaction are trophic and involve bacterial utilization or alteration of secreted fungal metabolites including organic acids. We hypothesize that sensation of fungal presence via metabolic signals initiates bacterial production of a second, uncharacterized signal that induces fungal growth. It is well known that fungi acidify their surroundings by the secretion of oxalates (Rosling et al., 2004; Stopnisek et al., 2016) and other organic acids. Several pathogenic fungi and bacteria sense and manipulate pH shifts in host immune cells enabling their persistence virulence (Prusky et al., 2004). Bacterial utilization of excreted fungal metabolites has been proposed to explain regular co-occurrence of *Burkholderia* and fungi (Stopnisek et al., 2016). However, to the best of our knowledge pH shifts associated with fungal fatty acids and their potential utilization as a trophic resource has not yet been considered signals in mutualistic BFIs.

Trophic trades are common in many symbioses between diverse organisms, particularly those involving bacterial endosymbionts (Wernegreen, 2002; Bianciotto et al., 2003; Nehls et al., 2007; Castillo and Pawlowska, 2010; Ghignone et al., 2012; Fujimura et al., 2014; Moné et al., 2014; Moran and Bennett, 2014; Su et al., 2014; Ainsworth et al., 2015; Uehling et al., 2017). Erythronic acid is a plant derived fatty acid with a well understood role in the establishment of nitrogen-fixing bacterial symbioses with plant hosts (Smith et al., 2015). In these systems the secretion of flavonoids and aldonic acids such as erythronic acid elicit the expression of NOD genes, comprising early inter-kingdom signaling events (Gagnon and Ibrahim, 1998). Caveats to consider include that many nitrogen fixing bacteria belong to Alphaproteobacteria and *Burkholderia* is a Betaproteobacterium, and we have not evaluated the presence or absence of NOD genes in the genome of the *Burkholderia* strain used in these experiments.

The nature of the second signal, which is propagated from bacteria to fungi after bacteria recognize fungal organic acids, remains to be identified. We observed that the second signal is conditionally expressed and remains in media after filtration, indicating that it is small, secreted, and potentially stable in solution at various pHs. The ubiquity of early symbiotic metabolic signal exchanges and its implications for the evolution of long-term, stable symbioses, especially for BFIs between Proteobacteria and Mucoromycota fungi is an exciting new avenue to be explored.

### Interactions Between Betaproteobacteria and Mucoromycota Share Commonalities

To explore the specificity of this interaction, we evaluated the ability of *Populus* rhizosphere isolates closely related to *Burkholderia* and *M. elongata* to initiate symbiotic growth increases. We quantified the effects of *Methylibium*–*M. elongata* and *Burkholderia*–*Umbelopsis* on each other’s growth rates using plate-based and optical density in liquid P20 co-culture. We found that *Methylibium* and *Umbelopsis* increased the fungal and bacterial growth rates and altered media pH in similar ways compared to *Burkholderia* and *M. elongata.* We concluded that the secretion of organic acids or other acidic metabolites may be a general signal for Betaproteobacterial detection of Mucoromycota fungi and induce growth stimulation and fungal signal production in *Burkholderia*. These data show that fungi in the Mucoromycota and bacteria in the Burkholderiales are prone to pre-symbiotic signaling.

The co-occurrence of *Burkholderia* and many diverse soil fungi has been shown to be partially trophic in nature, and it has been hypothesized to be enabled by conserved metabolic genomic content (Stopnisek et al., 2016). This idea is further exemplified by the ubiquity of several *Burkholderia* related fungal symbionts and their close relationships with Mucoromycota fungi (Bianciotto et al., 1996, 2003; Sato et al., 2010; Ghignone et al., 2012; Mondo et al., 2012, 2017; Lastovetsky et al., 2016; Ohshima et al., 2016; Uehling et al., 2017). The work presented here is novel in examining the extent of symbiotic phenotypes elicited by relatives of the microbes in question and demonstrating evolutionary conservation of interactions over small phylogenetic space.

Our results show that media conditioned by microbes is composed of numerous metabolites that shift in relative abundance correlated with microbial presence. However, our study is limited in that we did not evaluate how the addition of a single organic acid affects fungal or bacterial growth. We expect there to be multiple metabolic signals. Future directions will include profiling changes in gene expression in response to identified conditions and metabolic signals and evaluating the potential for symbiotic interactions between Betaproteobacteria and Mucoromycota to impact plant health.

## Conclusion

We investigated the dynamics of pre-symbiotic signaling in poplar-associated microbes *M. elongata* and *Burkholderia*. Our plate-based radial growth and optical density assays supported that bi-directional communication for growth stimulation occurs between these microbes. Further dissection of the interaction using time-lapse videography of co-cultures on agar plates found that hyphae at the edge of *M. elongata* colonies exhibit unique growth rates and behaviors, necessitating more precise fungal growth quantification methods. To compare fungal growth rates in various media, we used time-lapse microscopy leveraging a microfluidic device that we designed and fabricated.

By comparing fungal growth rates on agar plates, in the microfluidic devices, and in liquid cultures, we identified conditions that strongly affect microbial growth rates. The pre-symbiotic signaling was inferred to comprise multiple bi-directional signals resulting in bacterial utilization of fungal organic acids. Our current working model includes pre-symbiotic signaling that consists of at least two signaling events. The first is emission of fungal metabolites including fungal erythronic acid that are used or modified by bacteria. A second signal of unknown nature is produced by bacteria which induces fungal growth rates. Identifying the remaining signals exchanged, the nature of putative receptors involved, and the intracellular signal transduction will be the topics of future investigations.

## Materials and Methods

### Microbial Isolates and Conditioned Media Preparation

Microbial identity was confirmed as previously described, by sequencing the Internal Transcribed Spacer region (ITS) for fungi and 16S for bacteria (Uehling et al., 2017). Accession numbers for bacteria and fungi used in experiments are listed in Table 1. Microbes were grown in modified Pachlewski’s medium (Pachlewski, 1967) (P20) lacking thiamine, as previously described to promote BFIs (Di Battista et al., 1996; Deveau et al., 2007). Briefly, P20 is a modification of Pachlewski’s medium (Pachlewski, 1967) including 0.25 g di-ammonium tartrate, 0.5 g KH_2_PO_4_, 0.25 g MgSO_4_, 1 g glucose, 1 mL 1/10 diluted Kanieltra microelement solution and 20 g agar L^–1^. For media buffered at various pH, MES, and TRIS (Sigma-Aldrich) were added to liquid P20. To generate conditioned media (FSP, BFI, DC media), microbes were grown for 48 h in P20 media singly, in succession, or in co-culture as described in Table 3. For media conditioned by microbial growth (Table 3), stock cultures for fungal inocula were prepared by culturing *M. elongata* on solid P20 media and incubated at ambient temperature (19°C) without light until colony diameter was at least 5 cm (5 days). Fungal inocula were prepared by separating mycelia from the leading edge of the growing culture using a 1 cm diameter cork borer. Inoculation plugs were transferred to experimental cultures using a sterile stainless-steel spatula. The stock culture for bacterial inocula were prepared by transferring a single colony of *Burkholderia* to 10 mL of liquid P20 using a sterile inoculation loop. Bacterial cultures were incubated at 22°C in a Thermo Scientific MaxQ^TM^ 4000 shaker incubator at 100 rpm until exponential growth was observed (72 h). Growth stage was monitored by recording percent absorbance at 600 nm of a 1 mL aliquot of the bacterial inoculum every 24 h using a Thermo Scientific Genesys 20 spectrometer. After exponential growth was observed, the culture was diluted with P20 until OD600 was 0.8. Aliquots of 50 μL volume were used to inoculate experimental cultures. All experimental cultures and control cultures were grown in biological replicate (*n* = 4) in 10 mL P20 in sterile 15 mL conicals at 22°C in a Thermo Scientific MaxQ 4000 shaker incubator under gentle rotation (100 rpm). All filtrations were performed by pressing the full culture volume through a sterile 0.22 μm pore size syringe filter (Fisher) using a sterile, disposable syringe into a new conical tube. Filtrates for testing directional trophic interactions (DC media, Table 3 and Figure 3) were prepared by inoculating a liquid culture with fungi, growing for 48 h, filtering, inoculating with bacteria, incubating for 48 h, and then filtering again. Co-culture control filtrates (BFI, Table 3) were prepared by inoculating simultaneously with both bacteria and fungi in liquid P20 media, incubating for 48 h, and filtering. Axenic control filtrates (BSP/FSP, Table 3) were prepared by inoculating with either fungi or bacteria, incubating for 48 h, and filtering. All filtrates were frozen in liquid nitrogen and stored at −80°C prior to GC-MS analysis. *M. elongata* isolates AG77 with and without bacterial endosymbiont *Mycoavidus cysteinexigens* (CBS-KNAW Fungal Biodiversity Center accession number 137287), has been maintained in our laboratory. These isolates are referred to in Table 2 as AG77+/− where plus and minus refer to endosymbiont presence (Uehling et al., 2017).

### Microbial Growth Rate Quantification

For fungi, radial growth assays on agar plates were performed as previously described (Uehling et al., 2017). Briefly for each assay, a 5mm diameter plug inoculated with *M. elongata* was placed in the center of a plate. Bacteria were grown in P20 to 0.8 OD 600 and 50 μL were spotted equidistant at four corners around the fungal plug, at the edge of the plate. The assay was performed on two media MEA (nutrient rich) and P20 (nutrient poor). Colonies were traced at 48 h for both media. For bacterial growth curves, microbes were grown in 96-well flat-bottomed plates (Sigma-Aldrich), with 6 wells or replicates per treatment, and read on a Perkins Elmer Victor 3. 200 μL of conditioned media were inoculated with a premixed solution of media and bacteria at a 1:10 ratio, with the starting bacterial culture of OD600 = 0.8. Each well was covered with sterile mineral oil to avoid evaporation. Population dynamics were extrapolated from curve plotting using custom scripts in MATLAB (MathWorks). Colony growth rates were extrapolated from optical density readings that were recorded at a 600 nm wavelength (OD600) every 30 min for 50 h. Data were analyzed using custom scripts and visuals were prepared in MATLAB and edited using Photoshop (Adobe). Fungal growth rates derived from microfluidic devices were converted from μm/min to mm/day for comparisons of plate-based and microfluidic data.

### Microfluidic Device Design and Fabrication

In this device design (Figure 4), there are two microfluidic systems facing opposite directions per slide. Each microfluidic system consists of two inoculation ports, one round and the other square, facing each other. The ports are connected by four separate and parallel channels 50 mm in length, 100 μm in width and 5 μm in height (Figure 4). Four 5 μm wide narrow passages or “narrow necks” (NN) (Figures 4B,C) were implemented in the design to strain individual hyphae for time lapse video observation and quantification of growth rate shifts, based on previous observation that hyphal diameters of *M. elongata* are 5–10 μm (Uehling personal observation). The microfluidic devices were designed with precise rulers etched in 50, 100, and 500 μm intervals (Figures 4A–C), allowing accurate extrapolation of growth rates from time-lapse videos.

Microfluidic devices were designed and fabricated in house at Oak Ridge National Laboratory. Soda lime chrome masks were fabricated in house (Millet et al., 2015) and clean silicon wafers (Si specs) were used as starting material for microfluidic masters. Wafers were spin-coated with MicroPrime P20 adhesion promoter (Shin-Etsu MicroSi) to promote photo resist adhesion. NFR 016 D2 (JSR Micro, Inc., Sunnyvale, CA, United States), a negative-toned photoresist, was spin-coated onto the wafers (2 μm thickness), then soft baked (90°C, 90 s), exposed to 365-nm light for 3 s, and post-exposure baked at 115°C for 90 s. The resist pattern was developed for 1 min with Microposit^®^ MF^®^CD-26 developer (Shipley Company, Marlborough, MA, United States) then rinsed with deionized water and dried (nitrogen stream). The wafers were etched for 6 cycles using a modified Bosch process (3 s polymer deposition, 10 s etch) to produce a relief of the microfluidic channel with a profile height of 5.2–5.6 μm. Wafers were then treated with air plasma (2 min) immediately prior to silane exposure [trichloro (1H,1H,2H,2H-perfluoro-n-octyl) silane, 85°C, 60–120 min] to prevent polydimethylsiloxane (PDMS) adhesion to the microfluidic master during polymer cure.

Microfluidic channels were produced through a conventional soft lithography replication process using PDMS. The PDMS prepolymer and curing agent were mixed (10:1 ratio, respectively), then poured onto the wafer. To remove bubbles from the liquid-phase PDMS and surface of the master, the master with PDMS was placed into a vacuum chamber until bubbles were no longer visible. The PDMS polymer was cured on a level surface in an oven (70°C) for 2 h. Microfluidic systems were removed in pairs from the cured PDMS. Holes were punched in the PDMS using dermal biopsy punches; 5 mm hole punches were used for the inoculation ports. After punching out the ports for cell culture, the PDMS channels were air-plasma treated (2 min), placed on plasma-cleaned microscope slides, and placed in the curing oven (70°C, 20 min). After curing, the PDMS microfluidics on glass slides were sterilized by autoclaving (121°C, 20 min). Autoclaved microfluidics were transferred to sterile Petri dishes in a cell culture hood, then shipped and stored until used.

### Microfluidic Device Use, Imaging, and Validation

Each device was placed under house vacuum for 60 min in a sterile desiccator in a fume hood. 250 μL of media were then added to each device using sterile gel loading pipette tips. Media transit through devices was confirmed visually. *M. elongata* was grown for 2 days on MEA prior to device inoculations. Devices were inoculated with agar media plugs extracted from 2-day-old colonized MEA plates using a glass pipette tip to produce 1 × 3 mm diameter plugs. Fungi were grown and colonized chambers for 48 h in the desiccator, surrounded by a sterile water bath to minimize evaporation. Chamber colonization was confirmed visually with a light microscope before imaging. Microfluidic devices with microbes were imaged on an Axio Observer upright microscope with inverted camera at the Duke Light Microscopy Facility. Individual snaps were obtained every minute, and sewn together to make time-lapse videos in ImageJ (Abràmoff et al., 2004). Measurements are derived in units of μm per minute, and then converted to mm per day to compare rates between microfluidics and plates. Measurements of growth rates were performed in ImageJ, using the ruler etched in the PDMS device as a calibration for accuracy. Statistics analyses were performed in R (R Core Team, 2014).

To validate the microfluidic device design, the growth rates of *M. elongata* with (AG77 +) and without (AG77-) its bacterial endosymbiont, *M. cysteinexigens* were quantified. In previous work, we documented the growth increase in fungal isolates cleared of endosymbionts, and we recapitulated this result in our microfluidic device (Uehling et al., 2017) (Figure 4D and Table 2). Consistent with previous results (Uehling et al., 2017), the uncleared strain grew significantly slower than the cleared strain (4.75 ± 0.84 mm/day vs. 5.60 ± 0.99 mm/day) (Figures 3, 4D and Table 2), serving as a proof of principal for this device.

### Metabolite Quantification

For metabolomic profiling, media conditioned by microbial growth were obtained by culturing described above and 0.5-mL aliquots were dried in a nitrogen stream. Sorbitol was added (to achieve 15 ng/μL injected) before extraction as an internal standard to correct for differences in extraction efficiency due to subsequent differences in changes in sample volume during heating. Dried extracts were silylated as described previously (Li et al., 2012; Tschaplinski et al., 2012; Uehling et al., 2017) After 2 days, 1 μL aliquots were injected into an Agilent Technologies Inc. (Santa Clara, CA, United States) 5975C inert XL gas chromatograph-mass spectrometer, configured and operated as described earlier (Li et al., 2012; Tschaplinski et al., 2012; Uehling et al., 2017). Metabolite peaks were extracted using a key selected ion, characteristic m/z fragment, rather than the total ion chromatogram, to minimize integrating co-eluting metabolites. The extracted peaks of known metabolites were scaled back up to the total ion current using predetermined scaling factors. Peaks were quantified by area integration and the concentrations were normalized to the quantity of the internal standard (sorbitol) recovered, amount of sample extracted, derivatized, and injected. Metabolites of interest were identified using a large user-created database (>2300 spectra) of mass spectral electron impact ionization (EI) fragmentation patterns of trimethylsilyl-derivatized compounds and the Wiley Registry 10^th^ Edition with NIST 2014 mass spectral database.

## Data Availability Statement

The datasets generated for this study can be found in the NCBI KV442011.1, Go0012187, NZ_CP029606.1.

## Author Contributions

JU, JL, LM, CT, GB, JL, SR, TT, and RV conceived the experimental design. JL and MD provided the microbial cultures. JU, LM, JA, and SR designed and fabricated the microfluidics devices. JU, JL, ME, HM, NE, and TT generated and analyzed the data. JU, LM, and RV prepared and edited the manuscript. MD, SR, JWS, JES, TT, JL, and RV provided the project oversight and funding.

## Conflict of Interest

The authors declare that the research was conducted in the absence of any commercial or financial relationships that could be construed as a potential conflict of interest.

## Supplementary Material

FIGURE S1 Metabolite accumulation in P20 media by microbial conditioning. Metabolites are reported in ug/mL compared to sorbitol standards. Media conditioning is indicated by colored bars within each metabolite cluster for a media control (P20), fungal conditioned (FSP), bacterial conditioned (BSP), co-culture (BFI) or double conditioned (DC) media. See Table 3 for more detail on conditioning.

FIGURE S2 Metabolite accumulation in liquid P20 fungal conditioned (FSP) media compared to liquid P20 double conditioned (BFI) media, see Table 3 for detail on conditioning. The accumulation of metabolites arranged by category are reported as fold change in FSP relative to BFI media. Red bars indicate significance across replicates based on *p* < 0.05, Students *t*-test.

TABLE S1 Relative metabolite accumulation in media conditioned by microbial growth. Metabolites were quantified by gas chromatography-mass spectrometry and the resulting mass to charge ratios assigned to metabolites by comparing samples with internal sorbitol controls to public and custom mass spectral databases. Red values indicate a statistically significant accumulation of metabolites, blue values indicated a statistically significant decrease of metabolites. Conditions used for Figure 6 and Supplementary Figure S1 are indicated with gray backgrounds. Data for all conditions available on request.

VIDEO S1 Time lapse video of 6 h of *M. elongata* colony growth. The fungal colony on the left is growing toward the *Burkholderia* bacterial colony on the right.

VIDEO S2 Time lapse of 60 min of *M. elongata* hyphae growth in one of the chambers in the microfluidic device.
