# Supplementary figures and images for: Microfluidics and Metabolomics Reveal Symbiotic Bacterial–Fungal Interactions Between Mortierella elongata and Burkholderia Include Metabolite Exchange
Source: Front Microbiol. 2019 Oct 1;10:2163. doi: 10.3389/fmicb.2019.02163 (PMC6779839; doi:10.3389/fmicb.2019.02163)

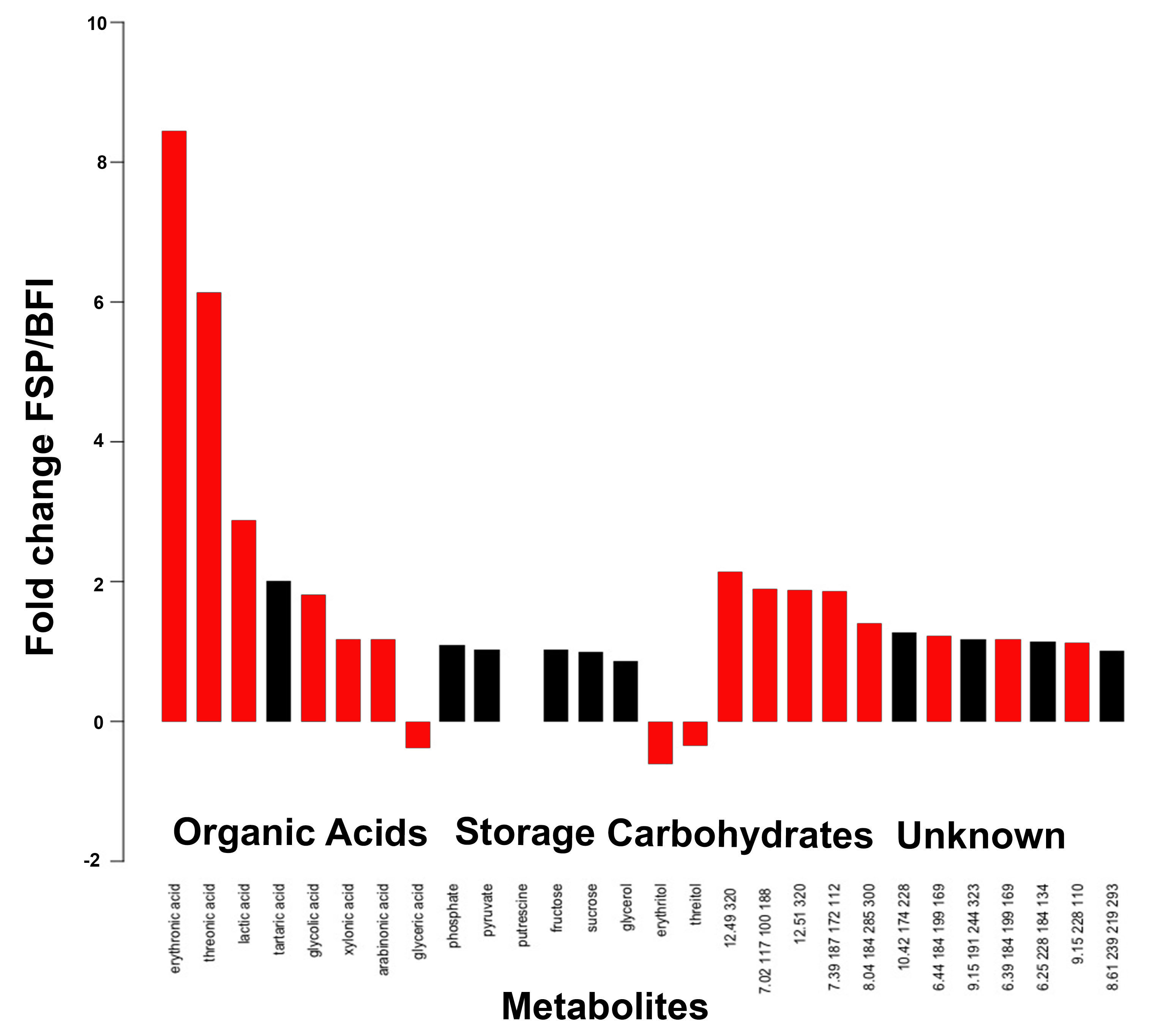

Supplement: FIGURE S1 — Metabolite accumulation in P20 media by microbial conditioning. Metabolites are reported in ug/mL compared to sorbitol standards. Media conditioning is indicated by colored bars within each metabolite cluster for a media control (P20), fungal conditioned (FSP), bacterial conditioned (BSP), co-culture (BFI) or double conditioned (DC) media. See Table 3 for more detail on conditioning. [file Image_1.JPEG]

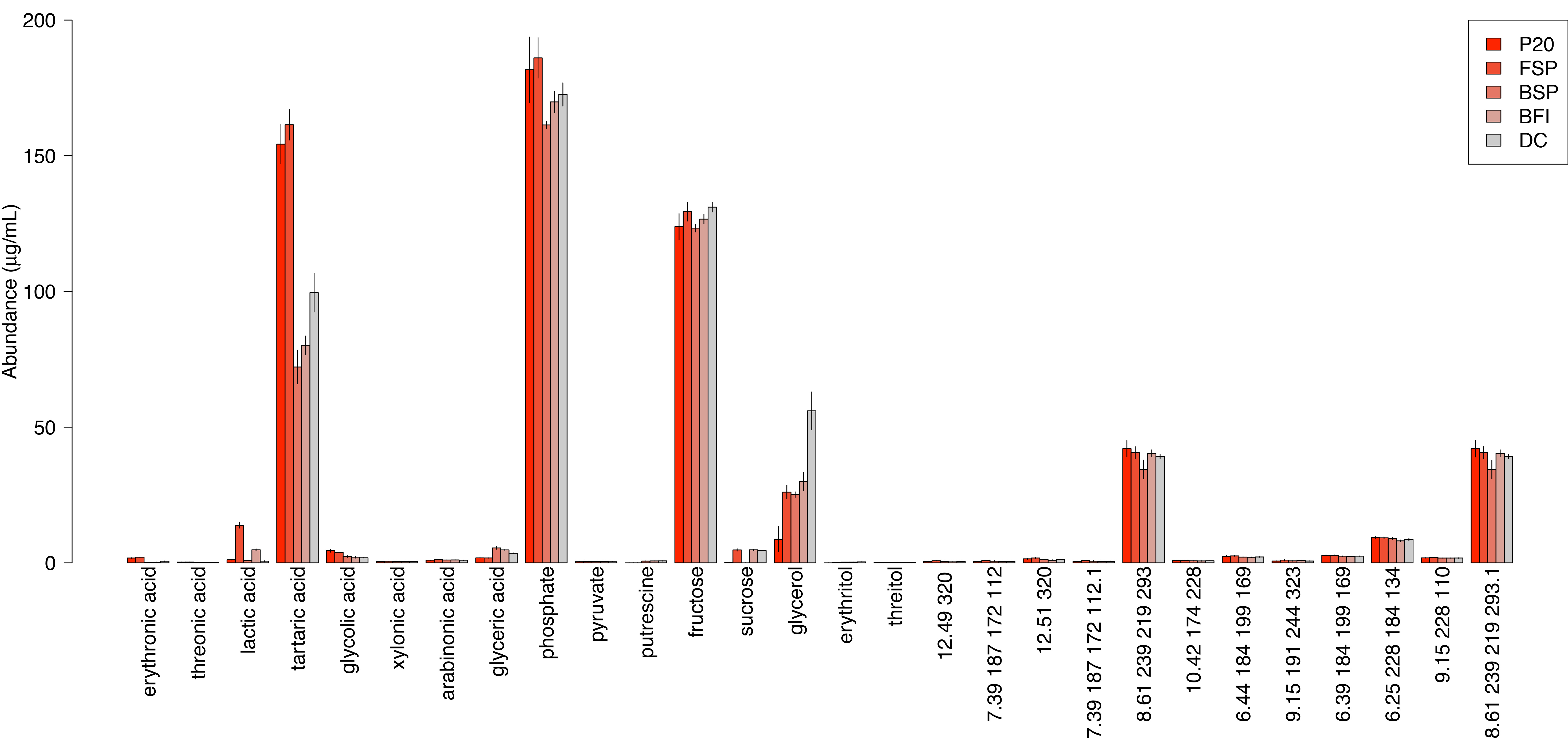

Supplement: FIGURE S2 — Metabolite accumulation in liquid P20 fungal conditioned (FSP) media compared to liquid P20 double conditioned (BFI) media, see Table 3 for detail on conditioning. The accumulation of metabolites arranged by category are reported as fold change in FSP relative to BFI media. Red bars indicate significance across replicates based on p < 0.05, Students t-test. [file Image_2.JPEG]
